# Supplementary material for: Pyruvate Dehydrogenase Kinase 1 inhibition mediated oxidative phosphorylation enhancement in cartilage promotes osteoarthritis progression
Source: BMC Musculoskelet Disord. 2023 Jul 20;24:597. doi: 10.1186/s12891-023-06585-6 (PMC10357736; doi:10.1186/s12891-023-06585-6)
Supplement: Supplementary file 1 — Additional file 1. [file 12891_2023_6585_MOESM1_ESM.zip › KEGG.Gsea.1653622432464/KEGG_CALCIUM_SIGNALING_PATHWAY.html]

Details for gene set KEGG\_CALCIUM\_SIGNALING\_PATHWAY[GSEA]

|  || Dataset | 55457\_collapsed\_to\_symbols.cls.cls #OA\_versus\_normal.cls.cls #OA\_versus\_normal\_repos |
| Phenotype | cls.cls#OA\_versus\_normal\_repos |
| Upregulated in class | normal |
| GeneSet | KEGG\_CALCIUM\_SIGNALING\_PATHWAY |
| Enrichment Score (ES) | -0.2222734 |
| Normalized Enrichment Score (NES) | -1.0478355 |
| Nominal p-value | 0.36070383 |
| FDR q-value | 0.58069426 |
| FWER p-Value | 1.0 |
Table: GSEA Results Summary

  

Fig 1: Enrichment plot: KEGG\_CALCIUM\_SIGNALING\_PATHWAY      
 Profile of the Running ES Score & Positions of GeneSet Members on the Rank Ordered List

  

| SYMBOL | TITLE | RANK IN GENE LIST | RANK METRIC SCORE | RUNNING ES | CORE ENRICHMENT || 1 | DRD1 | dopamine receptor D1 [Source:HGNC Symbol;Acc:HGNC:3020] | 392 | 0.709 | -0.0166 | No |
| 2 | SLC25A31 | solute carrier family 25 member 31 [Source:HGNC Symbol;Acc:HGNC:25319] | 395 | 0.708 | -0.0025 | No |
| 3 | HTR2B | 5-hydroxytryptamine receptor 2B [Source:HGNC Symbol;Acc:HGNC:5294] | 562 | 0.636 | -0.0028 | No |
| 4 | ADRB2 | adrenoceptor beta 2 [Source:HGNC Symbol;Acc:HGNC:286] | 596 | 0.625 | 0.0071 | No |
| 5 | PLCG2 | phospholipase C gamma 2 [Source:HGNC Symbol;Acc:HGNC:9066] | 725 | 0.588 | 0.0089 | No |
| 6 | PRKACB | protein kinase cAMP-activated catalytic subunit beta [Source:HGNC Symbol;Acc:HGNC:9381] | 771 | 0.576 | 0.0169 | No |
| 7 | RYR2 | ryanodine receptor 2 [Source:HGNC Symbol;Acc:HGNC:10484] | 828 | 0.561 | 0.0237 | No |
| 8 | PHKB | phosphorylase kinase regulatory subunit beta [Source:HGNC Symbol;Acc:HGNC:8927] | 853 | 0.554 | 0.0329 | No |
| 9 | AVPR1B | arginine vasopressin receptor 1B [Source:HGNC Symbol;Acc:HGNC:896] | 865 | 0.552 | 0.0431 | No |
| 10 | CAMK2A | calcium/calmodulin dependent protein kinase II alpha [Source:HGNC Symbol;Acc:HGNC:1460] | 916 | 0.538 | 0.0500 | No |
| 11 | ERBB2 | erb-b2 receptor tyrosine kinase 2 [Source:HGNC Symbol;Acc:HGNC:3430] | 963 | 0.526 | 0.0569 | No |
| 12 | CALM3 | calmodulin 3 [Source:HGNC Symbol;Acc:HGNC:1449] | 1039 | 0.510 | 0.0613 | No |
| 13 | GRIN2A | glutamate ionotropic receptor NMDA type subunit 2A [Source:HGNC Symbol;Acc:HGNC:4585] | 1054 | 0.506 | 0.0703 | No |
| 14 | PDE1A | phosphodiesterase 1A [Source:HGNC Symbol;Acc:HGNC:8774] | 1074 | 0.500 | 0.0788 | No |
| 15 | CALML5 | calmodulin like 5 [Source:HGNC Symbol;Acc:HGNC:18180] | 1261 | 0.465 | 0.0736 | No |
| 16 | ADCY7 | adenylate cyclase 7 [Source:HGNC Symbol;Acc:HGNC:238] | 1408 | 0.442 | 0.0710 | No |
| 17 | PTGFR | prostaglandin F receptor [Source:HGNC Symbol;Acc:HGNC:9600] | 1416 | 0.441 | 0.0792 | No |
| 18 | PLCE1 | phospholipase C epsilon 1 [Source:HGNC Symbol;Acc:HGNC:17175] | 1505 | 0.428 | 0.0809 | No |
| 19 | ADCY1 | adenylate cyclase 1 [Source:HGNC Symbol;Acc:HGNC:232] | 1589 | 0.415 | 0.0827 | No |
| 20 | RYR1 | ryanodine receptor 1 [Source:HGNC Symbol;Acc:HGNC:10483] | 1753 | 0.391 | 0.0778 | No |
| 21 | P2RX4 | purinergic receptor P2X 4 [Source:HGNC Symbol;Acc:HGNC:8535] | 1827 | 0.381 | 0.0797 | No |
| 22 | HTR2A | 5-hydroxytryptamine receptor 2A [Source:HGNC Symbol;Acc:HGNC:5293] | 1847 | 0.379 | 0.0858 | No |
| 23 | CCKAR | cholecystokinin A receptor [Source:HGNC Symbol;Acc:HGNC:1570] | 1849 | 0.379 | 0.0933 | No |
| 24 | GNA14 | G protein subunit alpha 14 [Source:HGNC Symbol;Acc:HGNC:4382] | 2187 | 0.340 | 0.0737 | No |
| 25 | PLCB4 | phospholipase C beta 4 [Source:HGNC Symbol;Acc:HGNC:9059] | 2217 | 0.337 | 0.0781 | No |
| 26 | PLCD1 | phospholipase C delta 1 [Source:HGNC Symbol;Acc:HGNC:9060] | 2298 | 0.327 | 0.0784 | No |
| 27 | BST1 | bone marrow stromal cell antigen 1 [Source:HGNC Symbol;Acc:HGNC:1118] | 2319 | 0.324 | 0.0833 | No |
| 28 | TNNC1 | "troponin C1, slow skeletal and cardiac type [Source:HGNC Symbol;Acc:HGNC:11943]" | 2396 | 0.315 | 0.0837 | No |
| 29 | CD38 | CD38 molecule [Source:HGNC Symbol;Acc:HGNC:1667] | 2416 | 0.312 | 0.0885 | No |
| 30 | TNNC2 | "troponin C2, fast skeletal type [Source:HGNC Symbol;Acc:HGNC:11944]" | 2417 | 0.312 | 0.0947 | No |
| 31 | SPHK2 | sphingosine kinase 2 [Source:HGNC Symbol;Acc:HGNC:18859] | 2478 | 0.305 | 0.0961 | No |
| 32 | CACNA1F | calcium voltage-gated channel subunit alpha1 F [Source:HGNC Symbol;Acc:HGNC:1393] | 2726 | 0.278 | 0.0823 | No |
| 33 | VDAC2 | voltage dependent anion channel 2 [Source:HGNC Symbol;Acc:HGNC:12672] | 2734 | 0.276 | 0.0873 | No |
| 34 | ATP2A1 | ATPase sarcoplasmic/endoplasmic reticulum Ca2+ transporting 1 [Source:HGNC Symbol;Acc:HGNC:811] | 2781 | 0.272 | 0.0891 | No |
| 35 | CAMK2G | calcium/calmodulin dependent protein kinase II gamma [Source:HGNC Symbol;Acc:HGNC:1463] | 2900 | 0.261 | 0.0851 | No |
| 36 | P2RX5 | purinergic receptor P2X 5 [Source:HGNC Symbol;Acc:HGNC:8536] | 2937 | 0.257 | 0.0874 | No |
| 37 | PDGFRA | platelet derived growth factor receptor alpha [Source:HGNC Symbol;Acc:HGNC:8803] | 2943 | 0.256 | 0.0921 | No |
| 38 | ATP2B2 | ATPase plasma membrane Ca2+ transporting 2 [Source:HGNC Symbol;Acc:HGNC:815] | 3023 | 0.247 | 0.0909 | No |
| 39 | ATP2B3 | ATPase plasma membrane Ca2+ transporting 3 [Source:HGNC Symbol;Acc:HGNC:816] | 3263 | 0.225 | 0.0766 | No |
| 40 | GRM1 | glutamate metabotropic receptor 1 [Source:HGNC Symbol;Acc:HGNC:4593] | 3313 | 0.220 | 0.0772 | No |
| 41 | ITPKB | inositol-trisphosphate 3-kinase B [Source:HGNC Symbol;Acc:HGNC:6179] | 3467 | 0.205 | 0.0693 | No |
| 42 | CHRM2 | cholinergic receptor muscarinic 2 [Source:HGNC Symbol;Acc:HGNC:1951] | 3473 | 0.205 | 0.0730 | No |
| 43 | TACR3 | tachykinin receptor 3 [Source:HGNC Symbol;Acc:HGNC:11528] | 3527 | 0.200 | 0.0729 | No |
| 44 | CACNA1I | calcium voltage-gated channel subunit alpha1 I [Source:HGNC Symbol;Acc:HGNC:1396] | 3642 | 0.190 | 0.0677 | No |
| 45 | CAMK4 | calcium/calmodulin dependent protein kinase IV [Source:HGNC Symbol;Acc:HGNC:1464] | 3664 | 0.189 | 0.0699 | No |
| 46 | PLN | phospholamban [Source:HGNC Symbol;Acc:HGNC:9080] | 3710 | 0.184 | 0.0700 | No |
| 47 | PLCB1 | phospholipase C beta 1 [Source:HGNC Symbol;Acc:HGNC:15917] | 3724 | 0.183 | 0.0727 | No |
| 48 | PRKCA | protein kinase C alpha [Source:HGNC Symbol;Acc:HGNC:9393] | 3728 | 0.183 | 0.0761 | No |
| 49 | PPP3CA | protein phosphatase 3 catalytic subunit alpha [Source:HGNC Symbol;Acc:HGNC:9314] | 3803 | 0.176 | 0.0738 | No |
| 50 | ADORA2A | adenosine A2a receptor [Source:HGNC Symbol;Acc:HGNC:263] | 3870 | 0.168 | 0.0720 | No |
| 51 | HTR2C | 5-hydroxytryptamine receptor 2C [Source:HGNC Symbol;Acc:HGNC:5295] | 3873 | 0.167 | 0.0752 | No |
| 52 | VDAC1 | voltage dependent anion channel 1 [Source:HGNC Symbol;Acc:HGNC:12669] | 3939 | 0.161 | 0.0733 | No |
| 53 | ATP2B4 | ATPase plasma membrane Ca2+ transporting 4 [Source:HGNC Symbol;Acc:HGNC:817] | 3998 | 0.156 | 0.0719 | No |
| 54 | P2RX2 | purinergic receptor P2X 2 [Source:HGNC Symbol;Acc:HGNC:15459] | 4029 | 0.153 | 0.0726 | No |
| 55 | CACNA1S | calcium voltage-gated channel subunit alpha1 S [Source:HGNC Symbol;Acc:HGNC:1397] | 4031 | 0.153 | 0.0756 | No |
| 56 | PTAFR | platelet activating factor receptor [Source:HGNC Symbol;Acc:HGNC:9582] | 4069 | 0.149 | 0.0757 | No |
| 57 | SLC25A6 | solute carrier family 25 member 6 [Source:HGNC Symbol;Acc:HGNC:10992] | 4211 | 0.136 | 0.0674 | No |
| 58 | ITPR2 | "inositol 1,4,5-trisphosphate receptor type 2 [Source:HGNC Symbol;Acc:HGNC:6181]" | 4222 | 0.135 | 0.0693 | No |
| 59 | NOS1 | nitric oxide synthase 1 [Source:HGNC Symbol;Acc:HGNC:7872] | 4228 | 0.134 | 0.0716 | No |
| 60 | P2RX7 | purinergic receptor P2X 7 [Source:HGNC Symbol;Acc:HGNC:8537] | 4415 | 0.120 | 0.0594 | No |
| 61 | CACNA1G | calcium voltage-gated channel subunit alpha1 G [Source:HGNC Symbol;Acc:HGNC:1394] | 4422 | 0.119 | 0.0613 | No |
| 62 | CHRNA7 | cholinergic receptor nicotinic alpha 7 subunit [Source:HGNC Symbol;Acc:HGNC:1960] | 4475 | 0.115 | 0.0595 | No |
| 63 | ADRA1A | adrenoceptor alpha 1A [Source:HGNC Symbol;Acc:HGNC:277] | 4556 | 0.109 | 0.0554 | No |
| 64 | GNA15 | G protein subunit alpha 15 [Source:HGNC Symbol;Acc:HGNC:4383] | 4595 | 0.105 | 0.0545 | No |
| 65 | EDNRA | endothelin receptor type A [Source:HGNC Symbol;Acc:HGNC:3179] | 4663 | 0.099 | 0.0513 | No |
| 66 | ERBB3 | erb-b2 receptor tyrosine kinase 3 [Source:HGNC Symbol;Acc:HGNC:3431] | 4863 | 0.083 | 0.0373 | No |
| 67 | GRM5 | glutamate metabotropic receptor 5 [Source:HGNC Symbol;Acc:HGNC:4597] | 4941 | 0.077 | 0.0328 | No |
| 68 | PPID | peptidylprolyl isomerase D [Source:HGNC Symbol;Acc:HGNC:9257] | 5029 | 0.071 | 0.0274 | No |
| 69 | SLC25A4 | solute carrier family 25 member 4 [Source:HGNC Symbol;Acc:HGNC:10990] | 5073 | 0.067 | 0.0253 | No |
| 70 | MYLK | myosin light chain kinase [Source:HGNC Symbol;Acc:HGNC:7590] | 5079 | 0.066 | 0.0263 | No |
| 71 | GNAL | G protein subunit alpha L [Source:HGNC Symbol;Acc:HGNC:4388] | 5158 | 0.059 | 0.0213 | No |
| 72 | ADCY9 | adenylate cyclase 9 [Source:HGNC Symbol;Acc:HGNC:240] | 5191 | 0.057 | 0.0200 | No |
| 73 | ADRA1B | adrenoceptor alpha 1B [Source:HGNC Symbol;Acc:HGNC:278] | 5275 | 0.050 | 0.0144 | No |
| 74 | MYLK3 | myosin light chain kinase 3 [Source:HGNC Symbol;Acc:HGNC:29826] | 5294 | 0.048 | 0.0140 | No |
| 75 | PPP3CB | protein phosphatase 3 catalytic subunit beta [Source:HGNC Symbol;Acc:HGNC:9315] | 5311 | 0.046 | 0.0137 | No |
| 76 | DRD5 | dopamine receptor D5 [Source:HGNC Symbol;Acc:HGNC:3026] | 5659 | 0.019 | -0.0132 | No |
| 77 | LHCGR | luteinizing hormone/choriogonadotropin receptor [Source:HGNC Symbol;Acc:HGNC:6585] | 5679 | 0.017 | -0.0144 | No |
| 78 | AGTR1 | angiotensin II receptor type 1 [Source:HGNC Symbol;Acc:HGNC:336] | 6122 | -0.014 | -0.0488 | No |
| 79 | BDKRB2 | bradykinin receptor B2 [Source:HGNC Symbol;Acc:HGNC:1030] | 6276 | -0.026 | -0.0603 | No |
| 80 | CALML3 | calmodulin like 3 [Source:HGNC Symbol;Acc:HGNC:1452] | 6348 | -0.032 | -0.0652 | No |
| 81 | CACNA1C | calcium voltage-gated channel subunit alpha1 C [Source:HGNC Symbol;Acc:HGNC:1390] | 6367 | -0.033 | -0.0660 | No |
| 82 | PHKG1 | phosphorylase kinase catalytic subunit gamma 1 [Source:HGNC Symbol;Acc:HGNC:8930] | 6451 | -0.039 | -0.0717 | No |
| 83 | PRKCB | protein kinase C beta [Source:HGNC Symbol;Acc:HGNC:9395] | 6453 | -0.039 | -0.0710 | No |
| 84 | PTGER1 | prostaglandin E receptor 1 [Source:HGNC Symbol;Acc:HGNC:9593] | 6527 | -0.044 | -0.0759 | No |
| 85 | ATP2A3 | ATPase sarcoplasmic/endoplasmic reticulum Ca2+ transporting 3 [Source:HGNC Symbol;Acc:HGNC:813] | 6559 | -0.046 | -0.0774 | No |
| 86 | ADCY8 | adenylate cyclase 8 [Source:HGNC Symbol;Acc:HGNC:239] | 6646 | -0.053 | -0.0831 | No |
| 87 | TBXA2R | thromboxane A2 receptor [Source:HGNC Symbol;Acc:HGNC:11608] | 6651 | -0.053 | -0.0823 | No |
| 88 | ITPR1 | "inositol 1,4,5-trisphosphate receptor type 1 [Source:HGNC Symbol;Acc:HGNC:6180]" | 6752 | -0.059 | -0.0890 | No |
| 89 | PDE1C | phosphodiesterase 1C [Source:HGNC Symbol;Acc:HGNC:8776] | 6819 | -0.064 | -0.0929 | No |
| 90 | ERBB4 | erb-b2 receptor tyrosine kinase 4 [Source:HGNC Symbol;Acc:HGNC:3432] | 6830 | -0.065 | -0.0923 | No |
| 91 | PDGFRB | platelet derived growth factor receptor beta [Source:HGNC Symbol;Acc:HGNC:8804] | 6914 | -0.073 | -0.0974 | No |
| 92 | CYSLTR2 | cysteinyl leukotriene receptor 2 [Source:HGNC Symbol;Acc:HGNC:18274] | 6965 | -0.077 | -0.0998 | No |
| 93 | PPP3CC | protein phosphatase 3 catalytic subunit gamma [Source:HGNC Symbol;Acc:HGNC:9316] | 7031 | -0.082 | -0.1032 | No |
| 94 | PLCB2 | phospholipase C beta 2 [Source:HGNC Symbol;Acc:HGNC:9055] | 7052 | -0.084 | -0.1031 | No |
| 95 | PLCB3 | phospholipase C beta 3 [Source:HGNC Symbol;Acc:HGNC:9056] | 7118 | -0.090 | -0.1064 | No |
| 96 | GRPR | gastrin releasing peptide receptor [Source:HGNC Symbol;Acc:HGNC:4609] | 7361 | -0.107 | -0.1233 | No |
| 97 | GNAQ | G protein subunit alpha q [Source:HGNC Symbol;Acc:HGNC:4390] | 7518 | -0.120 | -0.1331 | No |
| 98 | OXTR | oxytocin receptor [Source:HGNC Symbol;Acc:HGNC:8529] | 7534 | -0.121 | -0.1319 | No |
| 99 | CACNA1E | calcium voltage-gated channel subunit alpha1 E [Source:HGNC Symbol;Acc:HGNC:1392] | 7540 | -0.121 | -0.1298 | No |
| 100 | CACNA1H | calcium voltage-gated channel subunit alpha1 H [Source:HGNC Symbol;Acc:HGNC:1395] | 7635 | -0.129 | -0.1346 | No |
| 101 | CACNA1A | calcium voltage-gated channel subunit alpha1 A [Source:HGNC Symbol;Acc:HGNC:1388] | 7774 | -0.140 | -0.1427 | No |
| 102 | SLC25A5 | solute carrier family 25 member 5 [Source:HGNC Symbol;Acc:HGNC:10991] | 7877 | -0.146 | -0.1477 | No |
| 103 | PHKA1 | phosphorylase kinase regulatory subunit alpha 1 [Source:HGNC Symbol;Acc:HGNC:8925] | 7896 | -0.147 | -0.1462 | No |
| 104 | CHRM3 | cholinergic receptor muscarinic 3 [Source:HGNC Symbol;Acc:HGNC:1952] | 8034 | -0.158 | -0.1538 | No |
| 105 | HRH1 | histamine receptor H1 [Source:HGNC Symbol;Acc:HGNC:5182] | 8146 | -0.166 | -0.1592 | No |
| 106 | TACR2 | tachykinin receptor 2 [Source:HGNC Symbol;Acc:HGNC:11527] | 8160 | -0.168 | -0.1568 | No |
| 107 | ADRB1 | adrenoceptor beta 1 [Source:HGNC Symbol;Acc:HGNC:285] | 8212 | -0.172 | -0.1574 | No |
| 108 | PHKA2 | phosphorylase kinase regulatory subunit alpha 2 [Source:HGNC Symbol;Acc:HGNC:8926] | 8333 | -0.181 | -0.1632 | No |
| 109 | F2R | coagulation factor II thrombin receptor [Source:HGNC Symbol;Acc:HGNC:3537] | 8447 | -0.190 | -0.1682 | No |
| 110 | ADCY3 | adenylate cyclase 3 [Source:HGNC Symbol;Acc:HGNC:234] | 8572 | -0.199 | -0.1740 | No |
| 111 | CHP2 | calcineurin like EF-hand protein 2 [Source:HGNC Symbol;Acc:HGNC:24927] | 8573 | -0.199 | -0.1700 | No |
| 112 | PRKCG | protein kinase C gamma [Source:HGNC Symbol;Acc:HGNC:9402] | 8582 | -0.200 | -0.1666 | No |
| 113 | CAMK2B | calcium/calmodulin dependent protein kinase II beta [Source:HGNC Symbol;Acc:HGNC:1461] | 8782 | -0.215 | -0.1779 | No |
| 114 | GNAS | GNAS complex locus [Source:HGNC Symbol;Acc:HGNC:4392] | 8929 | -0.226 | -0.1849 | No |
| 115 | ATP2A2 | ATPase sarcoplasmic/endoplasmic reticulum Ca2+ transporting 2 [Source:HGNC Symbol;Acc:HGNC:812] | 9015 | -0.233 | -0.1869 | No |
| 116 | TACR1 | tachykinin receptor 1 [Source:HGNC Symbol;Acc:HGNC:11526] | 9090 | -0.239 | -0.1879 | No |
| 117 | CACNA1B | calcium voltage-gated channel subunit alpha1 B [Source:HGNC Symbol;Acc:HGNC:1389] | 9092 | -0.239 | -0.1832 | No |
| 118 | PDE1B | phosphodiesterase 1B [Source:HGNC Symbol;Acc:HGNC:8775] | 9159 | -0.245 | -0.1834 | No |
| 119 | ADORA2B | adenosine A2b receptor [Source:HGNC Symbol;Acc:HGNC:264] | 9255 | -0.252 | -0.1858 | No |
| 120 | GRIN2C | glutamate ionotropic receptor NMDA type subunit 2C [Source:HGNC Symbol;Acc:HGNC:4587] | 9515 | -0.275 | -0.2007 | No |
| 121 | ADCY2 | adenylate cyclase 2 [Source:HGNC Symbol;Acc:HGNC:233] | 9715 | -0.293 | -0.2104 | No |
| 122 | CACNA1D | calcium voltage-gated channel subunit alpha1 D [Source:HGNC Symbol;Acc:HGNC:1391] | 9867 | -0.308 | -0.2161 | Yes |
| 123 | VDAC3 | voltage dependent anion channel 3 [Source:HGNC Symbol;Acc:HGNC:12674] | 9878 | -0.310 | -0.2107 | Yes |
| 124 | SLC8A2 | solute carrier family 8 member A2 [Source:HGNC Symbol;Acc:HGNC:11069] | 9887 | -0.310 | -0.2051 | Yes |
| 125 | P2RX1 | purinergic receptor P2X 1 [Source:HGNC Symbol;Acc:HGNC:8533] | 9926 | -0.314 | -0.2018 | Yes |
| 126 | NOS3 | nitric oxide synthase 3 [Source:HGNC Symbol;Acc:HGNC:7876] | 9980 | -0.319 | -0.1995 | Yes |
| 127 | ADRA1D | adrenoceptor alpha 1D [Source:HGNC Symbol;Acc:HGNC:280] | 10031 | -0.325 | -0.1970 | Yes |
| 128 | PTGER3 | prostaglandin E receptor 3 [Source:HGNC Symbol;Acc:HGNC:9595] | 10073 | -0.328 | -0.1936 | Yes |
| 129 | TRPC1 | transient receptor potential cation channel subfamily C member 1 [Source:HGNC Symbol;Acc:HGNC:12333] | 10107 | -0.331 | -0.1896 | Yes |
| 130 | CALM1 | calmodulin 1 [Source:HGNC Symbol;Acc:HGNC:1442] | 10136 | -0.334 | -0.1850 | Yes |
| 131 | RYR3 | ryanodine receptor 3 [Source:HGNC Symbol;Acc:HGNC:10485] | 10344 | -0.355 | -0.1942 | Yes |
| 132 | ITPR3 | "inositol 1,4,5-trisphosphate receptor type 3 [Source:HGNC Symbol;Acc:HGNC:6182]" | 10390 | -0.360 | -0.1905 | Yes |
| 133 | HTR7 | 5-hydroxytryptamine receptor 7 [Source:HGNC Symbol;Acc:HGNC:5302] | 10399 | -0.360 | -0.1839 | Yes |
| 134 | ITPKA | inositol-trisphosphate 3-kinase A [Source:HGNC Symbol;Acc:HGNC:6178] | 10450 | -0.367 | -0.1805 | Yes |
| 135 | PRKACG | protein kinase cAMP-activated catalytic subunit gamma [Source:HGNC Symbol;Acc:HGNC:9382] | 10466 | -0.370 | -0.1742 | Yes |
| 136 | CALM2 | calmodulin 2 [Source:HGNC Symbol;Acc:HGNC:1445] | 10579 | -0.382 | -0.1754 | Yes |
| 137 | PRKACA | protein kinase cAMP-activated catalytic subunit alpha [Source:HGNC Symbol;Acc:HGNC:9380] | 10611 | -0.384 | -0.1701 | Yes |
| 138 | PLCG1 | phospholipase C gamma 1 [Source:HGNC Symbol;Acc:HGNC:9065] | 10722 | -0.395 | -0.1708 | Yes |
| 139 | CHRM5 | cholinergic receptor muscarinic 5 [Source:HGNC Symbol;Acc:HGNC:1954] | 10725 | -0.396 | -0.1630 | Yes |
| 140 | PHKG2 | phosphorylase kinase catalytic subunit gamma 2 [Source:HGNC Symbol;Acc:HGNC:8931] | 10830 | -0.408 | -0.1630 | Yes |
| 141 | CCKBR | cholecystokinin B receptor [Source:HGNC Symbol;Acc:HGNC:1571] | 10842 | -0.410 | -0.1557 | Yes |
| 142 | CHP1 | calcineurin like EF-hand protein 1 [Source:HGNC Symbol;Acc:HGNC:17433] | 10940 | -0.423 | -0.1548 | Yes |
| 143 | BDKRB1 | bradykinin receptor B1 [Source:HGNC Symbol;Acc:HGNC:1029] | 10951 | -0.424 | -0.1471 | Yes |
| 144 | HTR6 | 5-hydroxytryptamine receptor 6 [Source:HGNC Symbol;Acc:HGNC:5301] | 11034 | -0.435 | -0.1448 | Yes |
| 145 | GRIN2D | glutamate ionotropic receptor NMDA type subunit 2D [Source:HGNC Symbol;Acc:HGNC:4588] | 11306 | -0.472 | -0.1566 | Yes |
| 146 | PTK2B | protein tyrosine kinase 2 beta [Source:HGNC Symbol;Acc:HGNC:9612] | 11618 | -0.520 | -0.1706 | Yes |
| 147 | CYSLTR1 | cysteinyl leukotriene receptor 1 [Source:HGNC Symbol;Acc:HGNC:17451] | 11625 | -0.521 | -0.1606 | Yes |
| 148 | PRKX | protein kinase X-linked [Source:HGNC Symbol;Acc:HGNC:9441] | 11634 | -0.522 | -0.1508 | Yes |
| 149 | ATP2B1 | ATPase plasma membrane Ca2+ transporting 1 [Source:HGNC Symbol;Acc:HGNC:814] | 11648 | -0.524 | -0.1413 | Yes |
| 150 | ADRB3 | adrenoceptor beta 3 [Source:HGNC Symbol;Acc:HGNC:288] | 11691 | -0.531 | -0.1340 | Yes |
| 151 | HRH2 | histamine receptor H2 [Source:HGNC Symbol;Acc:HGNC:5183] | 11879 | -0.567 | -0.1373 | Yes |
| 152 | P2RX3 | purinergic receptor P2X 3 [Source:HGNC Symbol;Acc:HGNC:8534] | 11903 | -0.572 | -0.1276 | Yes |
| 153 | EDNRB | endothelin receptor type B [Source:HGNC Symbol;Acc:HGNC:3180] | 12075 | -0.613 | -0.1287 | Yes |
| 154 | EGFR | epidermal growth factor receptor [Source:HGNC Symbol;Acc:HGNC:3236] | 12109 | -0.622 | -0.1189 | Yes |
| 155 | SLC8A1 | solute carrier family 8 member A1 [Source:HGNC Symbol;Acc:HGNC:11068] | 12192 | -0.642 | -0.1124 | Yes |
| 156 | TRHR | thyrotropin releasing hormone receptor [Source:HGNC Symbol;Acc:HGNC:12299] | 12250 | -0.660 | -0.1037 | Yes |
| 157 | NOS2 | nitric oxide synthase 2 [Source:HGNC Symbol;Acc:HGNC:7873] | 12262 | -0.665 | -0.0912 | Yes |
| 158 | HTR4 | 5-hydroxytryptamine receptor 4 [Source:HGNC Symbol;Acc:HGNC:5299] | 12308 | -0.680 | -0.0811 | Yes |
| 159 | SPHK1 | sphingosine kinase 1 [Source:HGNC Symbol;Acc:HGNC:11240] | 12320 | -0.682 | -0.0683 | Yes |
| 160 | GNA11 | G protein subunit alpha 11 [Source:HGNC Symbol;Acc:HGNC:4379] | 12375 | -0.700 | -0.0585 | Yes |
| 161 | P2RX6 | purinergic receptor P2X 6 [Source:HGNC Symbol;Acc:HGNC:8538] | 12529 | -0.762 | -0.0552 | Yes |
| 162 | HTR5A | 5-hydroxytryptamine receptor 5A [Source:HGNC Symbol;Acc:HGNC:5300] | 12599 | -0.797 | -0.0447 | Yes |
| 163 | GRIN1 | glutamate ionotropic receptor NMDA type subunit 1 [Source:HGNC Symbol;Acc:HGNC:4584] | 12603 | -0.799 | -0.0289 | Yes |
| 164 | PPP3R1 | "protein phosphatase 3 regulatory subunit B, alpha [Source:HGNC Symbol;Acc:HGNC:9317]" | 12653 | -0.829 | -0.0161 | Yes |
| 165 | NTSR1 | neurotensin receptor 1 [Source:HGNC Symbol;Acc:HGNC:8039] | 12677 | -0.849 | -0.0009 | Yes |
| 166 | AVPR1A | arginine vasopressin receptor 1A [Source:HGNC Symbol;Acc:HGNC:895] | 12751 | -0.913 | 0.0117 | Yes |
Table: GSEA details [plain text format]

  

Fig 2: KEGG\_CALCIUM\_SIGNALING\_PATHWAY      
 Blue-Pink O' Gram in the Space of the Analyzed GeneSet

  

Fig 3: KEGG\_CALCIUM\_SIGNALING\_PATHWAY: Random ES distribution      
 Gene set null distribution of ES for **KEGG\_CALCIUM\_SIGNALING\_PATHWAY**
